# Pangenotypic and Genotype-Specific Antivirals in the Treatment of HCV Genotype 4 Infected Patients with HCV Monoinfection and HIV/HCV Coinfection

**DOI:** 10.3390/jcm11020389

**Published:** 2022-01-13

**Authors:** Dorota Zarębska-Michaluk, Jerzy Jaroszewicz, Anna Parfieniuk-Kowerda, Małgorzata Pawłowska, Ewa Janczewska, Hanna Berak, Justyna Janocha-Litwin, Jakub Klapaczyński, Krzysztof Tomasiewicz, Anna Piekarska, Rafał Krygier, Jolanta Citko, Olga Tronina, Krystyna Dobrowolska, Robert Flisiak

**Affiliations:** 1 Department of Infectious Diseases, Jan Kochanowski University Kielce, 25-516 Kielce, Poland; 2 Department of Infectious Diseases and Hepatology, Medical University of Silesia in Katowice, 40-055 Katowice, Poland; jerzy.jr@gmail.com; 3 Department of Infectious Diseases and Hepatology, Medical University of Białystok, 15-540 Białystok, Poland; anna.parfieniuk@gmail.com; 4 Department of Infectious Diseases and Hepatology, Ludwik Rydygier Collegium Medicum, Bydgoszcz Faculty of Medicine Nicolaus Copernicus University in Toruń, 85-030 Bydgoszcz, Poland; mpawlowska@cm.umk.pl; 5 Faculty of Health Sciences in Bytom, Department of Basic Medical Sciences, Medical University of Silesia, ID Clinic, Hepatology Outpatient Department, 41-902 Bytom, Poland; e.janczewska@poczta.fm; 6 Hospital for Infectious Diseases in Warszawa, 02-091 Warszawa, Poland; hberak@wp.pl; 7 Department of Infectious Diseases and Hepatology, Medical University Wrocław, 50-367 Wrocław, Poland; justynajanocha@o2.pl; 8 Department of Internal Medicine and Hepatology, Central Clinical Hospital of the Ministry of Internal Affairs and Administration, 02-507 Warszawa, Poland; klapaj@gmail.com; 9 Department of Infectious Diseases, Medical University of Lublin, 20-059 Lublin, Poland; tomaskdr@poczta.fm; 10 Department of Infectious Diseases and Hepatology, Medical University of Łódź, 90-419 Łódź, Poland; annapiekar@gmail.com; 11 Outpatients Hepatology Department, State University of Applied Sciences, 62-510 Konin, Poland; rafalkrygier@gmail.com; 12 Medical Practice of Infections, Regional Hospital, 10-561 Olsztyn, Poland; citkoj@wss.olsztyn.pl; 13 Department of Transplantation Medicine, Nephrology, and Internal Diseases, Medical University of Warsaw, 02-006 Warszawa, Poland; olgatronina@wp.pl; 14 Collegium Medicum, Jan Kochanowski University, 25-516 Kielce, Poland; krystyna.dobrowolska98@gmail.com

**Keywords:** hepatitis C virus, genotype 4, human immunodeficiency virus, direct-acting antivirals, pangenotypic, genotype-specific

## Abstract

The introduction of the direct-acting antivirals (DAA) has substantially improved the effectiveness of the therapy in patients with chronic hepatitis C. We aimed to compare the efficacy of pangenotypic and genotype-specific DAA in the cohort of genotype (GT) four patients with HCV monoinfection and HIV coinfection. A total of 662 GT4-infected patients treated in 2015–2020—of whom 168 (25.3%) were coinfected with HIV, selected from the retrospective EpiTer-2 database—were enrolled in the analysis. Among HIV-coinfected patients, 54% (90) were treated with genotype-specific regimens and 46% (78) with pangenotypic options, while among HCV-monoinfected patients, the rates were 72% and 28%, respectively. Significantly higher rate of males (67.9% vs. 57.7%, *p* = 0.01), a lower rate of liver cirrhosis (10.2% vs. 18.1%, *p* = 0.02), and higher of treatment-naïve patients (87.5% vs. 76.7%, *p* = 0.003) were documented in the HIV coinfected population. The overall sustained virologic response after exclusion of non-virologic failures was achieved in 98% with no significant difference between HIV-positive and HIV-negative patients, 96.2% vs. 98.5%, respectively. While the genotype-specific regimens resulted in a similar cure rate regardless of the HIV status, the pangenotypic options were more efficacious in patients with HCV monoinfection (99.3% vs. 94.4%, *p* = 0.05). Hereby, we demonstrated the high effectiveness and good safety profile of the DAA therapy in the population of HCV GT4 infected patients with HIV coinfection supporting the current recommendations to treat HCV/HIV coinfected patients with the same options as those with HCV monoinfection.

## 1. Introduction

The hepatitis C virus (HCV)—belonging to the genus Hepacivirus of the family Flaviviridae—is a small, enveloped, single-stranded, positive-sense ribonucleic acid (RNA) virus. Due to the wide genetic diversity of the viral genome, six major HCV genotypes (GT) with more than 30% difference in RNA sequence were described [1].

The most prevalent worldwide is GT1, accounting for nearly 50% of all cases of HCV, followed by the GT3 responsible for 30% of infections; while GT4 with an overall rate of 8% is the third by frequency [1]. The GT1 and GT3 infections dominate in most countries globally irrespective of the economic status, whereas the GT4 is more common in low-income countries. The highest prevalence of GT4 is documented in Sub-Saharan and North Africa and the Middle East [1]. The frequency of GT4 infections in Europe varies across countries; however, a growing prevalence has been observed in recent years in the south of the continent in the Mediterranean Sea region due to immigration [2,3]. An increasing share of GT4 has also been documented in Europe in people who inject drugs and patients coinfected with human immunodeficiency virus (HIV) [2,4,5].

In the interferon (IFN) era, the effectiveness of the antiviral therapy with pegylated IFN (pegIFN) and ribavirin (RBV) in GT4 was higher than achieved in the GT1 infected patients and lower compared to those with GT2 and GT3 infections [6]. The introduction of the first-class protease inhibitors, telaprevir (TVR) and boceprevir (BOC), used in combination with pegIFN and RBV has turned the situation into a disadvantage since these new drugs were active only in GT1-infected patients [7]. The next generation of direct-acting antivirals—protease inhibitor simeprevir (SMV), polymerase inhibitor sofosbuvir (SOF), and inhibitor of non-structural protein (NS) 5A daclatasvir (DCV)—were registered to use with the combination of pegIFN and RBV in GT4 infected patients with significant improvement in sustained virologic response (SVR) [8,9,10].

However, IFN-containing therapy in patients with coinfection with human immunodeficiency virus (HIV) was more difficult compared to those with HCV monoinfection. Difficulties in therapy resulted not only from lower response rates, but also from barriers in treatment use including the comorbidities, limited life expectancy, unfavorable safety profile, and non-adherence to therapy [11].

The real breakthrough in the treatment of GT4 infection was the implementation of direct-acting antivirals (DAA) [12]. The higher efficacy, substantially better tolerability, and shorter treatment duration broke down limitations associated with HIV-coinfection and has enabled wider access to antiviral therapy and eradicated HCV in this subpopulation. Genotype-specific regimens for GT4-infected patients, including ombitasvir/paritaprevir/ritonavir (OPr) + RBV, sofosbuvir/ledipasvir (SOF/LDV) ± RBV, and grazoprevir/elbasvir (GZR/ELB) ± RBV resulted in high treatment efficacy. Therefore, the first available pangenotypic options—SOF + RBV and SOF + DCV ± RBV—were infrequently used in this population [13]. The application of pangenotypic regimens in GT4 patients has increased with the advent of new drug formulations, glecaprevir/pibrentasvir (GLE/PIB) and sofosbuvir/velpatasvir (SOF/VEL) ± RBV. Genotype-specific regimens have been available in Poland since mid-2015, pangenotypic therapies have been available since mid-2018 and there have been no restrictions on access to therapy for HIV coinfected patients.

Clinical trials on DAA regimens evaluating efficacy and safety in HCV/HIV coinfected population have demonstrated high cure rates and good tolerablity relative to IFN-based therapies; however, data on the real-world effectiveness of DAA, especially pangenotypic regimens, in HIV-positive patients with GT4 infection are limited [14,15,16,17,18]. Therefore, the current analysis aimed to evaluate the IFN-free treatment in the cohort of GT4 patients with HIV coinfection considering the type of therapy used.

## 2. Materials and Methods

### 2.1. Study Design and Materials

**Figure 1:**
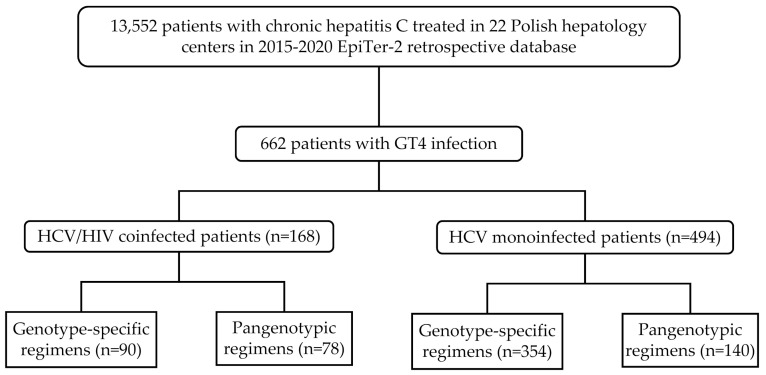
Flow chart showing the selection and stratification of patients included in the study.

The patients included in the current analysis were selected from the EpiTer-2 database (of 13,552 patients treated for chronic hepatitis C in 2015–2020 (Figure 1), (http://www.pteilchz.org.pl/informacje/epiter-2/ assessed on 28 December 2021). This is an ongoing large retrospective multicenter national real-world study evaluating DAA-based antiviral treatment in individuals with chronic hepatitis C treated in 22 Polish hepatology centers. The selection of the antiviral regimen was made by the treating physicians based on the medical knowledge according to the national recommendations of the Polish Group of Experts for HCV and the principles of the reimbursed therapeutic program established by the National Health Fund (NHF) considering the potential drug–drug interactions with antiretroviral therapy (ART) [19]. Drug doses and treatment duration were consistent with the Summary of Product Characteristics.

Patients provided informed consent before the start of the treatment according to the requirements of NHF. Patients’ data were collected retrospectively and submitted via a web platform operated by Tiba sp. z o.o. following the National General Data Protection Regulation in Poland.

The information captured at baseline included demographic and clinical data: age, gender, body mass index (BMI), comorbidities and concomitant medications, the severity of liver disease, hepatitis B virus (HBV) and human immunodeficiency virus (HIV) coinfections, and the history of previous antiviral treatment.

The degree of liver disease was evaluated noninvasively by the transient elastography (TE) or shear-wave elastography (SWE), or histologically by the liver biopsy. The measurement of the liver stiffness was a basis for assigning the patient to the fibrosis stage F0-F4 according to the METAVIR score using the recommendations of the European Association for the Study of the Liver (EASL) with 13 kilopascals as a threshold to define liver cirrhosis [20]. The cirrhotic patients were evaluated for the presence of esophageal varices and scored in the Child–Pugh (CP) scale and Model of End-Stage Liver Disease (MELD).

Characteristics of the patients at the start of the therapy also included laboratory data such as the activity of the serum alanine transaminase (ALT); the concentration of the bilirubin, albumin, creatinine, and hemoglobin; white blood cell and platelet counts; and HCV viral load.

The HCV RNA was assessed at baseline, at the end of treatment, and at least 12 weeks after therapy completion.

The assessment of the viral load was performed by the real-time polymerase chain reaction assays and genotyping was performed using reverse hybridization assays.

### 2.2. Efficacy and Safety Evaluation

The primary efficacy outcome was the sustained virologic response (SVR) defined as the negative result of HCV RNA 12 weeks after treatment. The secondary outcome was the effectiveness of the therapy depending on the HIV status and type of regimen—genotype-specific versus pangenotypic.

Genotype-specific regimens include OPr + RBV, GZR/EBR, and SOF/LDV, while the pangenotypic regimens were as follows: SOF ± DCV ± RBV, SOF/VEL ± RBV, and GLE/PIB.

The safety of the therapy was assessed by the rate of the treatment course modification and discontinuation, the prevalence of adverse events (AE) including the severe AE, and deaths. The AE of particular interest associated with the deterioration of the liver function such as ascites, encephalopathy, and gastrointestinal bleeding were assessed in patients with liver cirrhosis.

The intention-to-treat group (ITT) included patients who received at least one dose of antiviral drug, and the per-protocol group (PP) was established by excluding patients because of non-virologic failure.

### 2.3. Statistical Analysis

Results were expressed as mean ± standard deviation (SD) and median with 10–90% confidence intervals or number (percentage). A *p*-value of less than 0.05 was considered significant. The significance of differences was calculated by the χ2 or Fisher exact tests for nominal variables and by the Mann–Whitney test and the Kruskal–Wallis analysis of variance for continuous variables. For outcome analyses, odds ratios with 95% confidence intervals were additionally calculated. Univariable comparisons were calculated using the GraphPad Prism 5.1 software (GraphPad Software, Inc., La Jolla, CA, USA). 

## 3. Results

### 3.1. Characteristics of the Study Population

A total of 662 patients with HCV GT4 infection with mean age 45.3 ± 12.6 years, male predominance (60.3%), and 15.9% rate of liver cirrhosis treated in 2015–2020 were enrolled in the analysis (Figure 1). Most of them were treatment-naïve (79.5%) and the most commonly used DAA regimen in the current therapy was OPr + RBV (39.6%). One hundred sixty-eight patients (25.3%) were coinfected with HIV and 162 of them (96.4%) were on the antiretroviral therapy with the most common regimen (121; 74.7%) consisted of emtricitabine and tenofovir alafenamide (109 patients) or disoproxil (12 patients) followed by the abacavir and lamivudine-based option (22; 13.6%). In the remaining 19 patients, other therapeutic options were used.

A significantly higher rate of males (67.9% vs. 57.7%, *p* = 0.01) and a higher percentage of concomitant medications (97.6% vs. 52.2%, *p* < 0.001) were documented in HCV/HIV co-infected compared to patients with HCV monoinfection, while monoinfected individuals were more frequently diagnosed with comorbidities (59.5% vs. 38.1%, *p* < 0.001), the statistically significant difference was found for arterial hypertension and kidney diseases (*p* < 0.001) (Table 1).

The rate of patients with liver cirrhosis was significantly higher in the HCV monoinfected population (18.1% vs. 10.2%). The detailed characteristics regarding the severity of liver disease and HBV coinfection depending on the HIV status are presented in Table 2.

The higher rate of treatment-naïve patients (87.5% vs. 76.7%) was demonstrated in HIV-positive compared to HIV-negative individuals (Table 3).

In those with failure of the previous antiviral therapy, the pegylated interferon and ribavirin was the most common option used in both subpopulations. The genotype-specific regimen of OPr + RBV was more commonly used in HCV monoinfected patients, 42.9% vs. 29.8%, *p* = 0.003. Pangenotypic options were significantly more frequently applied in HIV-positive patients, 46.4% vs. 28.3%, *p* = 0.003 in HCV monoinfected individuals.

Of the pangenotypic regimens, the combination of SOF/VEL ± RBV was significantly more common in HCV/HIV-coinfected (24.4% vs. 13.6%, *p* = 0.002), while the absolute difference in GLE/PIB application was not statistically significant (20.2% vs. 14.2%, *p* = 0.07).

### 3.2. Efficacy

The overall sustained virologic response according to ITT analysis was 94.9%, but after exclusion of non-virologic failures, it reached 98% with no significant difference between HCV/HIV coinfected and HCV monoinfected patients, 96.2% vs. 98.5% respectively. A significant difference in the SVR rate was found when the comparison was performed according to ITT analysis due to a higher percentage of lost to follow-up patients among HCV/HIV-coinfected individuals, 91.7% vs. 96%, *p* = 0.04.

The analysis considering the type o regimen used revealed that genotype-specific regimens resulted in a comparable SVR rate regardless of the HIV status, 97.7% of HIV-coinfected and 98.3% of HCV-monoinfected individuals responded to therapy, *p* = 0.67 in the PP analysis. In the case of pangenotypic regimens, the HIV-negative patients responded in a higher percentage than HIV-positive, 99.3% vs. 94.4%, with the difference on the verge of statistical significance in the PP analysis (*p* = 0.05) and significant in ITT analysis (*p* = 0.007) (Table 4).

**Figure 2:**
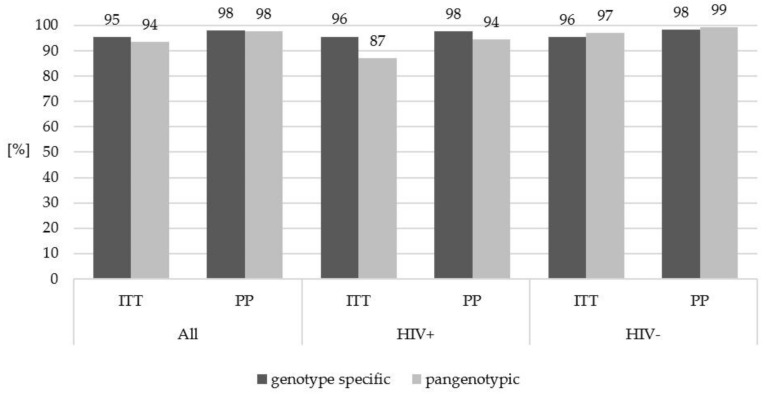
Treatment effectiveness in HIV-positive and HIV-negative patients according to the type of therapeutic regimen used. HIV, human immunodeficiency virus; ITT, intent to treat; PP, per protocol. No statistically significant differences were found between the analyzed groups.

In the evaluation carried out in the subpopulations of patients considering the type of therapy, no significant difference in the treatment efficacy with genotype-specific versus pangenotypic options was found in either HIV negative or HIV positive individuals (Figure 2).

Thirteen virologic non-responders, six HCV/HIV-coinfected and seven HCV monoinfected, were documented (Table 5).

Five of them were females, three patients were diagnosed with liver cirrhosis, including two scoring as A and one as B on the CP scale. Four treatment failures were previously treated with IFN-based regimens, three received pegIFN + RBV and one simeprevir + pegIFN + RBV. One of six HIV-coinfected nonresponders was not treated with ART while three were on emtricitabine + tenofovir alafenamide, two of them with cobicistat and elvitegravir and one with darunavir, two remaining HIV positive failures received abacavir + lamivudine and dolutegravir. All patients who did not respond to therapy completed the treatment course according to schedule and nine were negative for HCV RNA at the end of therapy.

No significant differences in demographic, clinical, and laboratory parameters were found in virologic failures compared to responders (Supplementary Table S1).

Comparative analysis by HIV status showed no statistically significant differences between responders and virologic non-responders in both HCV monoinfection and HIV coinfection groups.

The analysis of the SVR in specific subpopulations regarding gender, BMI, presence of the liver fibrosis F4, and the history of IFN-free therapy is presented in Table 6.

The majority of patients completed therapy according to schedule (95.8%) no impact of HIV-coinfection was observed (Supplementary Table S2).

### 3.3. Safety

Treatment modification in the form of reduction or discontinuation of the RBV was documented in seven patients. In one HIV-negative individual therapy was discontinued due to liver decompensation. At least one AE was documented in 23% of patients, the most common were weakness/fatigue and anemia noticed significantly more frequently in HCV monoinfected patients (0.01% vs. 5.1%, *p* = 0.02). One death due to non-liver cancer in monoinfected population and three serious AE were documented during the treatment course and follow-up period.

## 4. Discussion

The overlapping transmission routes resulted in a high prevalence of HCV/HIV coinfection. It is estimated that approximately 25% of patients living with HIV are coinfected with HCV worldwide. However, the prevalence rates vary significantly across subpopulations and geographic regions [21,22].

The introduction of the DAA options meant that this population is now treated equally to those infected with HCV only. To the best of our knowledge, the analyzed cohort is the most numerous population of HCV GT4 patients with HCV/HIV coinfection who have undergone DAA therapy, including pangenotypic regimens, to date. It should be emphasized that we have not confirmed the progression of hepatic fibrosis and more severe liver disease in this coinfected population, as reported by other researchers [23]. On the contrary, the rate of cirrhotic patients among GT4 infected HIV-positive population was significantly lower compared to that with HCV GT4 monoinfection, and any coinfected patient with liver cirrhosis presented hepatic decompensation at baseline. Furthermore, none of the coinfected individuals in the analyzed cohort had a history of hepatocellular carcinoma or liver transplantation.

A significantly higher percentage of treatment-naïve patients in the HIV-positive group in the current study confirms that in the era of DAA they gained wider access to antiviral therapy compared to the previous period, which is supported by the data from the literature [11].

The current study demonstrated the overall high efficacy of the DAA treatment in HCV/HIV coinfected patients, not different from the effectiveness obtained in the group of those with HCV GT4 monoinfection. Our data on the comparable SVR rate regardless of the HIV status support results from numerous clinical trials with the usage of different DAA regimens in HIV positive GT4 infected patients [14,15,16,17,18,24]. In groups of 5 to 30 HCV GT4/HIV-coinfected participants of C-EDGE CO-INFECTION (GZR/EBR), ASTRAL-5 (SOF/VEL), TURQOISE-I (OPr ± DSV ± RBV), EXPEDITION-2 (GLE/PIB), ION-4 (SOF/LDV), and HEPNED-001 studies, effectiveness of 93–100% following therapy with different DAA regimens was reported [14,15,16,17,18,24].

Similar efficacy was documented in real-world studies of HCV/HIV coinfection conducted by Machado et al., Piekarska et al., Bischoff et al., Navarro et al., and Minosse et al., but only some of them included a larger group of GT4 infected patients (33–77 participants) [25,26,27,28,29]. In the abovementioned real-world analyses, patients were treated predominantly with genotype-specific regimens, only a few patients received the first available pangenotypic option of SOF ± DCV ± RBV.

Even a real-world study of the largest cohort of HIV-positive patients with HCV GT4 infection to date conducted by Berenguer et al. did not evaluate the new pangenotypic regimens [30]. Of the 530 such patients accounting for 22.4% of all analyzed HCV/HIV coinfected Spanish population, 34% were diagnosed with liver cirrhosis. They were treated almost exclusively with two genotype-specific regimens, SOF/LDV and OPr and the cure rates in modified ITT analysis were 95.5% and 94.7% in patients without cirrhosis, and 93.9% and 100% in those with cirrhosis, respectively. Twenty patients with decompensated liver cirrhosis receiving SOF/LDV achieved a significantly lower SVR of 80%. One GT4 HCV/HIV-coinfected patient was treated with SOF + DCV and another one with SOF + SMV, both achieved an SVR [30].

Another real-world study was conducted by Sousa et al. in Spain on just one DAA regimen of OPr ± RBV with or without dasabuvir covering a group of 2408 HCV patients of whom 386 patients were HIV coinfected showed that infection with GT4 was associated with non-response to treatment in the multivariate analysis [31].

The current study documented an SVR rate of 98% for genotype-specific regimens regardless of the HIV status, while the effectiveness of the pangenotypic options (almost exclusively GLE/PIB and SOF/VEL combinations) was lower among GT4 HCV/HIV coinfected individuals and did not reach 95% as compared to 99% in HCV monoinfected subpopulation. Due to the lack of real-world studies evaluating new pangenotypic options in HIV coinfected GT4 HCV patients, clinical trials EXPEDITION-2 and ASTRAL-5 remain the only point of reference [15,17]. In both studies, the SVR was achieved by all HIV coinfected GT4 participants, but the small number of such patients should be highlighted, 17 and 5 individuals, respectively. One of the possible explanations for this discrepancy could be a relatively high rate of non-virologic nonresponders among HIV coinfected patients treated with pangenotypic regimens in our analysis, 7% compared to 2% in the genotype-specific arm. It can be assumed that the negative results in these lost-to-follow-up patients could affect the final effectiveness. All four virologic nonresponders to pangenotypic regimens were treated with the combination of GLE/PIB which is an unexpected finding requiring further analysis in the larger population. No possible drug–drug interactions between the ART and DAAs were expected, all of them were on the emtricitabine/tenofovir alafenamide, two received also cobicistat and elvitegravir. They were treated according to the label of GLE/PIB for 8 weeks, all of them were treatment-naïve and diagnosed with liver fibrosis F1–F2.

The likely reason for the higher efficacy of genotype specific therapy in HIV co-infected GT4-infected patients is that it had been used before when most patients waited in line to access new interferon-free treatment options, and thus showed greater cure determination and adherence. Unfortunately, our study did not assess adherence, so we cannot confirm this hypothesis. Moreover, HCV reinfection during the post-treatment follow-up period in these patients cannot be ruled out since such a phenomenon was demonstrated previously [32,33].

There were no safety issues during DAA therapy in GT4 HCV/HIV coinfected patients, no hepatic decompensation and no death were reported and only one serious AE not related to DAA treatment was documented, the frequency of AEs was comparable to HCV monoinfected patients. It should be highlighted that the selection of the therapeutic regimen was made upon the baseline evaluation of the ART to avoid the possible drug–drug interactions which could result in worse tolerability. Our findings on the favorable safety profile are in accordance with numerous clinical trials and real-world studies conducted in patients with HIV/HCV coinfection [15,17,26,30,34,35].

There are some limitations of the current study that should be considered when interpreting the results. One of them is observational design and retrospective data collecting with possible DAA selection bias, which is a result of real-world investigator decision study design. Some potentially useful data are missing, including drug monitoring and objective adherence records; also, the adverse events may be underreported in real-world settings. Another limitation is the relatively small sample sizes of HIV coinfected patients in the context of single treatment DAA regimens, especially DAA-failures among whom only three had liver cirrhosis and four previous anti-HCV failure preventing the performance of multivariate analysis. Finally, no baseline resistance mutation testing was performed.

However, the main strength of the current analysis is the largest number of HIV/HCV GT4 coinfected patients treated with DAA using both genotype-specific and pangenotypic regimens, to date. This multicenter analysis covers a heterogeneous population, representative of the real-world study. We use a population of HCV GT4 monoinfected patients as a comparator group.

## 5. Conclusions

We confirmed the high effectiveness and good safety profile of the DAA therapy in the population of HCV GT4 infected patients with HIV coinfection supporting the current recommendations to treat HCV/HIV coinfected patients with the same options as those with HCV monoinfection. The comparative analysis considering the type of regimen used documented the lower SVR achieved by those treated with pangenotypic options. Further studies are needed to allow the accumulation of non-responders to determine the actual outcomes of HCV GT4 treatment with pangenotypic regimens in HIV co-infected.

## Figures and Tables

**Table 1:** Baseline characteristics of GT4-infected patients depending on the HIV status.

Parameter	HIV+	HIV−	*p* =
	n = 168	n = 494	
Gender, females/males, n (%)	54 (32.1)/114 (67.9)	209 (42.2)/285 (57.7)	0.01
Age (years) mean (SD)	43.3 (7.7)	45.9 (13.8)	0.11
Median (10–90%CI)	43 (34–53)	44 (28–64)	
BMI mean (SD)	25.2 (3.9)	26.0 (4.6)	0.19
Median (10–90%CI)	25.3 (20.2–30.3)	25.3 (20.6–32.4)	
Any comorbidity, n (%)	64 (38.1)	294 (59.5)	<0.001
Arterial hypertension	15 (8.9)	149 (30.2)	<0.001
Diabetes	6 (3.6)	40 (8.1)	0.05
Autoimmune diseases	1 (0.6)	15 (3)	0.08
Kidney diseases	2 (1.2)	48 (9.7)	<0.001
incl. kidney transplantation	0	29 (5.9)	<0.001
Non-HCC tumors	1 (0.6)	7 (1.4)	0.67
Depression	8 (4.8)	25 (5.1)	1
Other	52 (31)	184 (37.2)	0.16
Concomitant medications, n (%)	164 (97.6)	258 (52.2)	<0.001
ALT IU/L, mean (SD)	70 (94)	71 (59)	0.01
Median (10–90%CI)	45 (22–135)	53 (22–140)	
Bilirubin mg/dL, mean (SD)	0.7 (0.5)	0.7 (0.5)	0.02
Median (10–90%CI)	0.5 (0.2–1.1)	0.6 (0.3–1.4)	
Albumin g/dL, mean (SD)	4.1 (0.5)	4.1 (0.5)	0.36
Median (10–90%CI)	4.1 (3.5–4.6)	4.1 (3.5–4.7)	
Creatinine mg/dL, mean (SD)	0.9 (0.2)	1.1 (1.2)	0.01
Median (10–90%CI)	0.9 (0.6–1.1)	0.8 (0.6–1.2)	
Hemoglobin g/dL, mean (SD)	14.7 (1.7)	14.5 (1.8)	0.4
Median (10–90%CI)	14.7 (12.5–16.9)	14.7 (12.2–16.6)	
Platelets, ×1000/µL, mean (SD)	210 (86)	200 (71)	0.4
Median (10–90%CI)	209 (123–284)	201 (101–286)	
HCV RNA × 10^6^ IU/mL, mean (SD)	2.4 (3.4)	2.72 (9.55)	0.45
Median (10–90%CI)	0.9 (0.05–7.8)	1.1 (0.1–5.6)	

GT, genotype; HIV, human immunodeficiency virus; SD, standard deviation; BMI, body mass index; non-HCC, non-hepatocellular carcinoma; ALT, alanine transaminase; HCV RNA, ribonucleic acid of hepatitis C virus.

**Table 2:** Characteristics of the liver disease in GT4-infected patients depending on the HIV status.

Parameter	HIV+	HIV−	*p* =
	n = 168	n = 494	
Liver fibrosis, n (%)			
F0	4 (2.4)	9 (1.8)	
F1	88 (52.4)	241 (48.9)	
F2	39 (23.2)	92 (18.6)	0.23
F3	18 (10.7)	55 (11.1)	
F4	17 (10.1)	88 (17.8)	
no data	2 (1.2)	9 (1.8)	
F0–F3	149 (89.8)	397 (81.9)	0.02
F4	17 (10.2)	88 (18.1)	
History of hepatic decompensation, n (%)	1 (0.6)	7 (1.4)	0.69
Ascites	0	6 (1.2)	
Ascites+encephalopathy	1 (0.6)	0	
Encephalopathy	0	1 (0.2)	
Documented esophageal varices, n (%)	1 (0.6)	29 (5.9)	0.002
Hepatic decompensation at baseline, n (%)	0	5 (1)	0.33
Ascites	0	3 (0.6)	
Encephalopathy	0	2 (0.4)	
HCC history, n (%)	0	5 (1)	0.33
OLTx history, n (%)	0	3 (0.6)	0.57
Child–Pugh, n (%)			
B	1 (0.6)	12 (2.4)	0.2
C	0	0	
HBV coinfection (HBsAg+), n (%)	3 (1.8)	7 (1.4)	0.72

GT, genotype; HIV, human immunodeficiency virus; HCC, hepatocellular carcinoma; OLTx, orthotopic liver transplantation; HBV, hepatitis B virus; HBsAg, hepatitis B surface antigen.

**Table 3:** Comparison of characteristics in GT4-infected patients depending on the HIV status.

Parameter	HIV+	HIV−	*p* =
	n = 168	n = 494	
History of previous therapy, n (%)			
Treatment-naïve	147 (87.5)	379 (76.7)	
Nonresponder	9 (5.3)	58 (11.8)	
Relapser	6 (3.6)	20 (4)	0.03
Discontinuation due to safety reason	4 (2.4)	15 (3)	
Unknown type of response	2 (1.2)	22 (4.5)	
Treatment-naïve	147 (87.5)	379 (76.7)	0.003
Treatment-experienced	21 (12.5)	115 (23.3)	
Previous regimen in treatment-experienced, n (%)	n = 21	n = 115	
IFN	0	6 (5.2)	0.59
PegIFN + RBV	15 (71.4)	93 (80.8)	0.38
SOF + PegIFN + RBV	0	1 (0.9)	1
SMV + PegIFN + RBV	0	6 (5.2)	0.59
SOF + RBV	1 (4.8)	0	0.15
SOF/LDV ± RBV	0	3 (2.6)	1
OPr + RBV	1 (4.8)	3 (2.6)	0.49
GZR/EBR	1 (4.8)	1 (0.9)	0.29
ASV + DCV	0	1 (0.9)	1
No data	3 (14.2)	1 (0.9)	0.01
Previous IFN-free regimen in patients with treatment failure, n (%)	n = 21	n = 115	
	3 (17.8)	8 (6.9)	0.37
Current treatment regimens, n (%)			
Genotype-specific			
OPr + RBV	50 (29.8)	212 (42.9)	0.003
GZR/EBR ± RBV	38 (22.6)	124 (25.1)	0.53
SOF/LDV ± RBV	2 (1.2)	18 (3.6)	0.12
Pangenotypic			
SOF + RBV	3 (1.8)	3 (0.6) *	0.17
GLE/PIB	34 (20.2)	70 (14.2)	0.07
SOF/VEL ± RBV	41 (24.4)	67 (13.6)	0.002
Current genotype-specific regimens, n (%)	90 (53.6)	354 (71.7)	<0.001
Current pangenotypic treatment regimens, n (%)	78 (46.4)	140 (28.3)	

HIV, human immunodeficiency virus; IFN, interferon; PegIFN, pegylated interferon; RBV, ribavirin; SOF, sofosbuvir; SMV, simeprevir; LDV, ledipasvir; OPr, ombitasvir, paritaprevir boosted ritonavir; GZR, grazoprevir; EBR, elbasvir; ASV, asunaprevir; DCV, daclatasvir; GLE, glecaprevir; PIB, pibrentasvir; VEL, velpatasvir. * One patient received a regimen with DCV.

**Table 4:** Treatment effectiveness following genotype-specific and pangenotypic regimens according to the HIV status.

		HIV+	HIV−	*p*	OR (95%CI)
All	ITT	154/168 (91.7)	474/494 (96.0)	0.04	0.46 (0.23–0.94)
	PP	154/160 (96.2)	474/481 (98.5)	0.10	0.38 (0.12–1.14)
Genotype-specific	ITT	86/90 (95.6)	338/354 (95.5)	1.00	1.02 (0.33–3.12)
	PP	86/88 (97.7)	338/344 (98.3)	0.67	0.76 (0.15–3.85)
Pangenotypic	ITT	68/78 (87.2)	136/140 (97.1)	0.007	0.20 (0.06–0.66)
	PP	68/72 (94.4)	136/137 (99.3)	0.05	0.12 (0.01–1.14)

HIV, human immunodeficiency virus; ITT, intent to treat; PP, per protocol; OR, odds ratio; CI, confidence interval.

**Table 5:** Characteristics of 13 virologic failures.

Patient	Age	HIV+	F, CP	Regimen	History of Previous Therapy	ART Regimen	Baseline HCV RNA IU/mL	Treatment Course Completed	EOT	Comment
Female 1	35	yes	1	GLE/PIB, 8 weeks	treatment-naive	abacavir, lamivudin, dolutegravir	2,700,000	according to schedule	TND	
Male 1	47	yes	1	OPr + RBV, 12 weeks	relapser (PR)	emtricitabin, tenofovir alafenamide	4,538,833	according to schedule	TD	
Male 2	52	yes	4, CP A	GZR/EBR, 12 weeks	treatment-naive	abacavir, lamivudin, dolutegravir	1,890,000	according to schedule	TND	
Male 3	28	yes	1	GLE/PIB, 8 weeks	treatment-naive	emtricitabin, tenofovir alafenamide, cobicistat, elvitegravir	473,000	according to schedule	TND	
Male 4	30	yes	2	GLE/PIB, 8 weeks	treatment-naive	emtricitabin, tenofovir alafenamide, cobicistat, elvitegravir	863,000	according to schedule	TND	
Male 5	44	yes	1	GLE/PIB, 8 weeks	treatment-naive	none	668,519	according to schedule	TD	
Female 2	46	no	1	SOF/VEL, 12 weeks	treatment-naive	na	542,000	according to schedule	TD	
Female 3	78	no	4, CP A	OPr+RBV, 12 weeks	null responder (PR)	na	1,378,790	according to schedule	TND	
Female 4	25	no	1	GZR/EBR+RBV, 12 weeks	treatment-naive	na	3,561,961	according to schedule	TND	
Female 5	79	no	2	GZR/EBR, 12 weeks	treatment-naive	na	91,900	according to schedule	TND	
Male 6	48	no	1	OPr+RBV, 12 weeks	relapser (PR + SMV)	na	3,600,000	according to schedule	TND	non-adherence
Male 7	46	no	4, CP B	LDV/SOF, 12 weeks	treatment-naive	na	317,281	according to schedule	TND	
Male 8	25	no	1	GZR/EBR, 12 weeks	null-responder (PR)	na	6,320,000	according to schedule	TD	

HIV, human immunodeficiency virus; F, fibrosis; CP, Child–Pugh scale; ART, antiretroviral; HCV RNA, ribonucleic acid of hepatitis C virus; EOT, end of treatment; GLE, glecaprevir; PIB, pibrentasvir; TND, target not detected; OPr, ombitasvir, paritaprevir boosted ritonavir; RBV, ribavirin; PR, Pegylated interferon + Ribavirin; TD, target detected; GZR, grazoprevir; EBR, elbasvir; SOF, sofosbuvir; VEL, velpatasvir; na, not applicable; SMV, simeprevir; LDV, ledipasvir.

**Table 6:** Treatment effectiveness in subpopulations.

	SVR ITT	SVR PP
Females, n = 263	252/263 (95.8)	252/257 (98.1)
Males, n = 399	376/399 (94.2)	376/384 (97.9)
IFN-free failure, n = 11	10/11 (91)	10/10 (100)
BMI > 30, n = 108	101/108 (93.5)	101/103 (98.1)
Fibrosis F4, n = 105	98/105 (93.3)	98/101 (97)

SVR, sustained virologic response; ITT, intent to treat; PP, per protocol; IFN, interferon; BMI, body mass index.

## Data Availability

Data supporting reported results can be provided upon request from the corresponding author.

## Supplementary Materials

The following supporting information can be downloaded at: https://www.mdpi.com/article/10.3390/jcm11020389/s1, Table S1: Virologic nonresponders versus responders among GT4-infected patients; Table S2: Safety of antiviral treatment in GT4-infected patients.
