# Supplementary material for: Pangenotypic and Genotype-Specific Antivirals in the Treatment of HCV Genotype 4 Infected Patients with HCV Monoinfection and HIV/HCV Coinfection
Source: J Clin Med. 2022 Jan 13;11(2):389. doi: 10.3390/jcm11020389 (PMC8781964; doi:10.3390/jcm11020389)
Supplement: Supplementary file 1 [file jcm-11-00389-s001.zip › jcm-1507008-supplementary.pdf]

Journal of
Clinical Medicine
MDPI

Suuplementary Table S1. Virologic nonresponders versus responders among GT4-infected patients

Parameter	Virologic nonresponders n=13	Responders n=628	p=
Gender, females/males, n(%)	5 (38.5) / 8 (61.5)	252 (40.1) / 376 (59.5)	1.00
Age [years] mean (SD)	44,8 (1.67)	45.3 (12.6)	0.61
BMI mean (SD)	25.1 (5.6)	25.8 (4.4)	0.20
Any comorbidity (except HBV and HIV coinfections), n(%)	8 (61.5)	329 (52.4)	0.58
Concomitant medications, n(%)	9 (69.2)	398 (63.4)	0.78
HBV coinfection (HBsAg+), n(%)	0	10 (1.6)	1.00
HIV coinfection, n(%)	6 (46.2)	154 (24.5)	0.10
Liver fibrosis F4, n(%)	3 (23.1)	98 (15.6)	0.44
History of hepatic decompensation, n(%)	0	7 (1.1)	1.00
HCC history, n(%)	0	4 (0.6)	1.00
Hepatic decompensation at baseline, n(%)	0	5 (0.8)	1.00
Child-Pugh B, n(%)	1 (7.7)	12 (1.9)	0.23
Treatment-experienced, n(%)	4 (30.8)	127 (20.2)	0.31
IFN-free DAA-experienced, n(%)	0	10 (1.6)	1.00
Current pangenotypic regimen, n(%)	5 (38.5)	204 (32.8)	0.77
ALT IU/L, mean (SD)	78 (42)	71 (71)	0.16
Bilirubin mg/dL, mean (SD)	0.8 (0.5)	0.7 (0.5)	0.42
Albumin g/dL, mean (SD)	3.9 (0.6)	4.1 (0.5)	0.37
Creatinine mg/dL, mean (SD)	0.9 (0.2)	1.0 (1.0)	0.14
Hemoglobin g/dL, mean (SD)	14.8 (1.8)	14.5 (1.8)	0.56
Platelets, x1000/μL, mean (SD)	182 (74)	202 (75)	0.28
HCV RNA x10^6 IU/ml, mean (SD)	2.07 (1.93)	2.68 (8.62)	0.54

GT, genotype; SD, standard deviation; BMI, body mass index; HBV, hepatitis B virus; HIV, human immunodeficiency virus; HBsAg, hepatitis B surface antigen; HCC, hepatocellular carcinoma; IFN, interferon; DAA, direct-acting antivirals; ALT, alanine transaminase; HCV RNA, ribonucleic acid of hepatitis C virus.

Supplementary Table S2. Safety of antiviral treatment in GT4-infected patients

Parameter	HIV+ n=168	HIV- n=494	p=
Treatment course, n(%)			
according to schedule	162 (96.4)	472 (95.5)	0.82
modified RBV dosage/RBV discontinuation	2 (1.2)	15 (3.1)	0.26
therapy discontinuation	2 (1.2)	5 (1)	1.00
no data	2 (1.2)	2 (0.4)	0.27
Patients with at least one AE	36 (21.4)	116 (23.5)	0.67
Most common AEs			
weakness/fatigue	19 (11.3)	60 (12.1)	0.89
anemia	2 (1.2)	25 (5.1)	0.02
Death	0	1 (0.2)	1.00
Other serious adverse events	1 (0.6)	2 (0.4)	1.00
AEs leading to treatment discontinuation	0	1 (0.2)	1.00
AEs of particular interest			
ascites	0	2 (0.4)	1.00

hepatic encephalopathy	0	0	-
gastrointestinal bleeding	0	1 (0.2)	1.00

GT, genotype; RBV, ribavirin; AE, adverse events.
Death – non-liver cancer
Other serious AE – HIV+ urinary tract infection, HIV- hydrothorax, icterus
AE leading to TD – decompensation
